# Cutaneous Intravascular CD-30-Positive Anaplastic Large Cell Lymphoma: A Case Report and Literature Review

**DOI:** 10.7759/cureus.44450

**Published:** 2023-08-31

**Authors:** Kiana Banafshay, Dylan Maldonado, Michelle Tarbox

**Affiliations:** 1 Department of Dermatology, Texas Tech University Health Sciences Center, Lubbock, USA

**Keywords:** anaplastic large cell lymphoma, cutaneous t-cell lymphoma, cd30 positive, t-cell lymphoma, alk negative

## Abstract

Intravascular cutaneous anaplastic large cell lymphoma (ALCL) is an extremely rare non-Hodgkin lymphoma that proliferates in the lumen of small blood vessels and has a propensity to manifest in the skin. Most reported cases of intravascular lymphoma described in the literature are of large B-cell lymphomas, making T-cell lymphomas incredibly rare. As such, we present the case of an 87-year-old male with primary cutaneous intravascular anaplastic large T-cell lymphoma that initially presented with an erythematous, subcutaneous nodule on the right mid-abdomen. We report the immunohistochemical results showing lymphoma cells staining positively for CD3 and CD30 and lacking expression of anaplastic lymphoma kinase, pan-cytokeratin, CD10, CD20, and SOX10. We also review and compare previously reported cases of intravascular ALCL with primary cutaneous involvement.

## Introduction

Cutaneous-anaplastic large cell lymphoma (C-ALCL) is a CD30-positive T-cell lymphoma within the subset of primary cutaneous CD30+ lymphoproliferative disorders with proposed criteria for diagnosis to include large, anaplastic, pleomorphic cells with a predominance of CD-30+ cells (>75%) and the absence of clinical evidence of mycosis fungoides [1]. Primary C-ALCL often presents as a solitary erythematous nodule limited to the skin and is anaplastic lymphoma kinase (ALK) negative, unlike the majority of systemic ALCL cases. We present a case of primary cutaneous, ALK-negative, anaplastic large T-cell lymphoma confined to the vascular spaces.

## Case presentation

**Figure 1:**
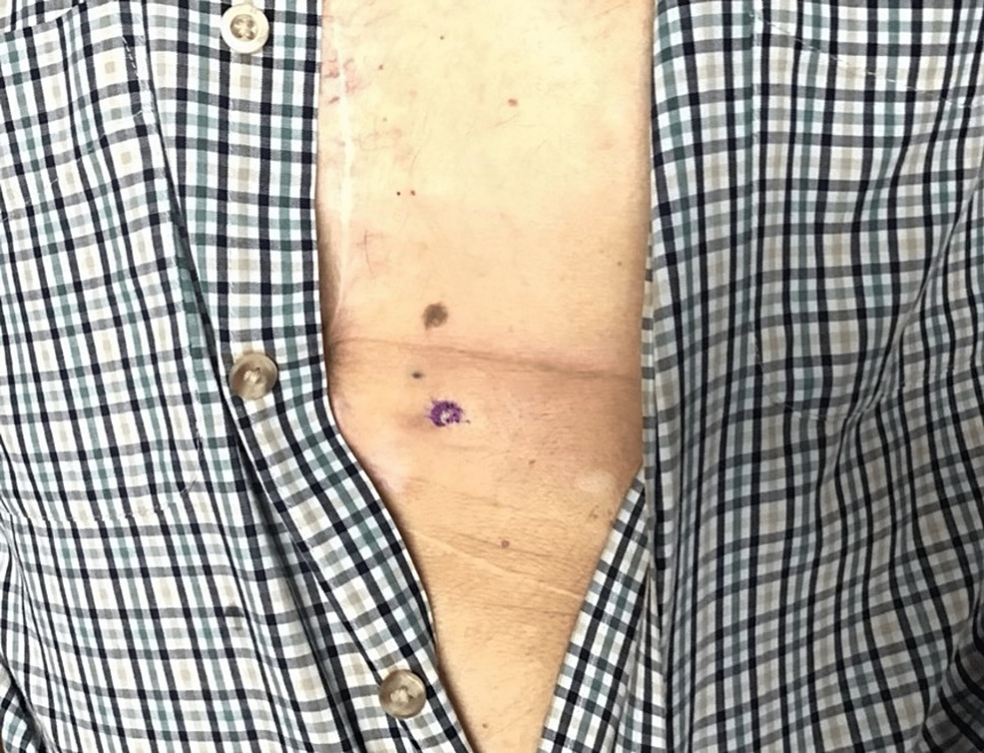
The patient’s initial presentation showing a firm, erythematous, subcutaneous nodule on the right mid-abdomen.

An 87-year-old male with a medical history of follicular non-Hodgkin lymphoma, treated over 10 years prior, presented to the clinic with a firm, erythematous, subcutaneous nodule on the right mid-abdomen (Figure 1). The patient’s medical history also included atrial fibrillation, hyperlipidemia, hypertension, coronary artery disease, and a previous diagnosis of malignant neoplasm of the esophagus.

**Figure 2:**
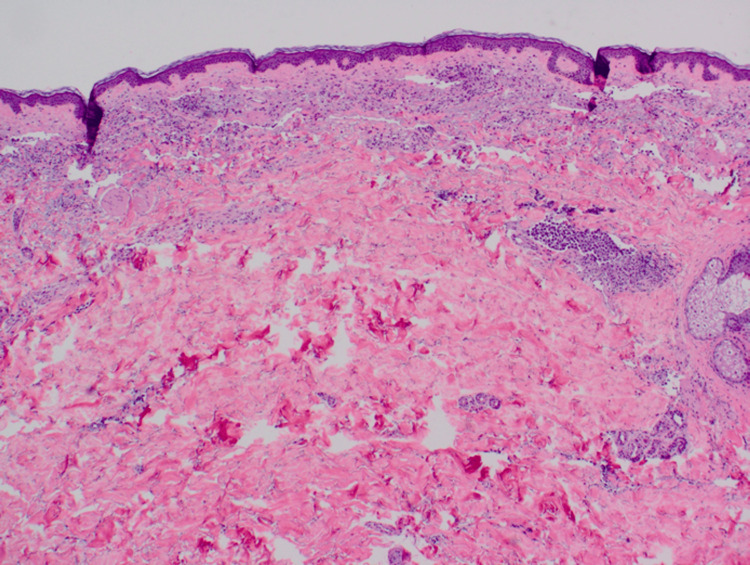
Low-power view showing scattered superficial perivascular infiltrate along with a lymphoid infiltrate composed of larger cells confined to vascular spaces within the dermis (20×).

**Figure 3:**
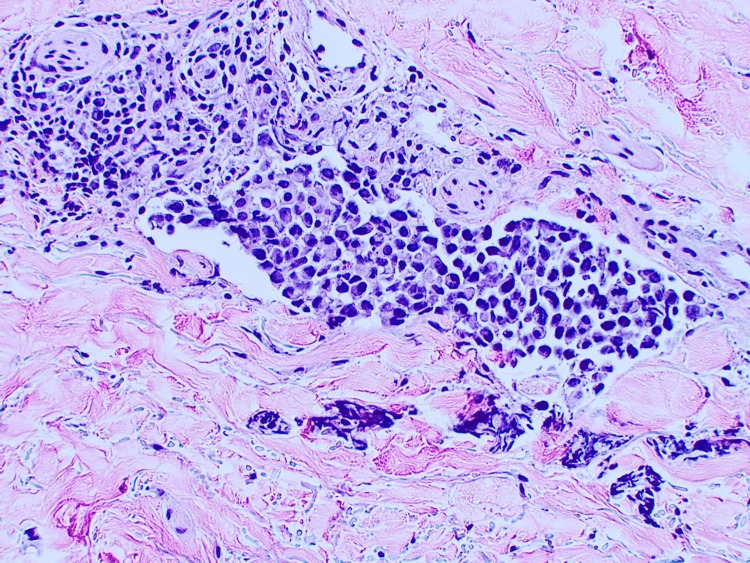
High-power view showing the intravascular atypical lymphocytes (100×).

**Figure 4:**
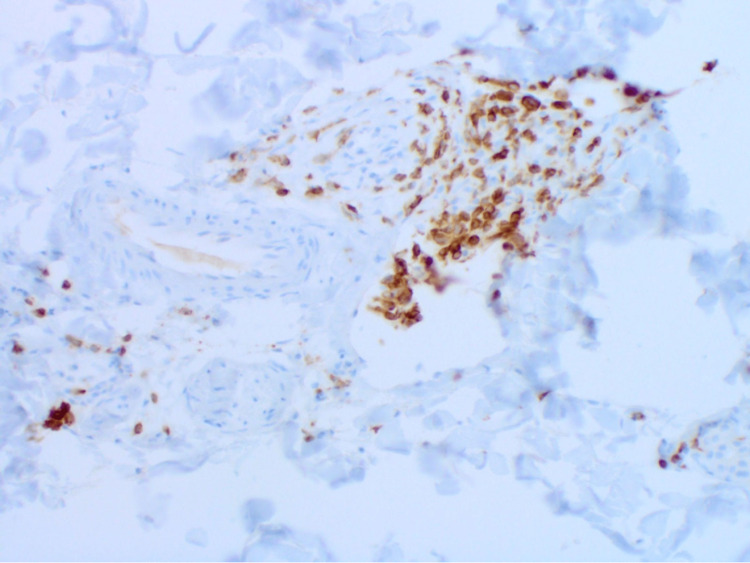
Presence of CD3-positive T cells confirmed (100×).

**Figure 5:**
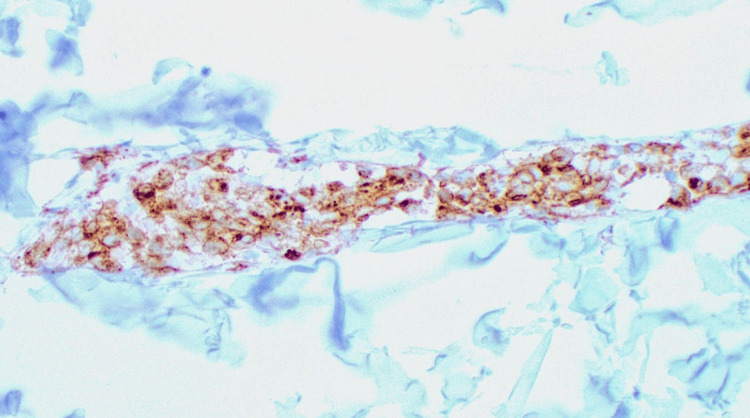
Presence of CD30-positive T cells confined to the vascular spaces (100×).

A review of the systems was negative. Initially, the clinical suspicion was that of an epidermal inclusion cyst. A small elliptical excision of the nodule was performed, and histopathology results revealed a superficial scant perivascular infiltrate of lymphocytes with an infiltrate of larger atypical cells in the deep dermis, which were confined to the vascular spaces (Figure 2). The large cells confined to the vascular spaces demonstrated marked cytologic atypia and frequent mitotic activity (Figure 3). Pan-cytokeratin, CD10, CD20, and SOX10 stains were negative in the intravascular cells. CD3 and CD30 were markedly positive (Figures 4, 5). In addition, there was scattered CD3 positivity of banal-appearing lymphocytes within the superficial dermis. The official pathological diagnosis of intravascular anaplastic large T-cell lymphoma was made.

**Figure 6:**
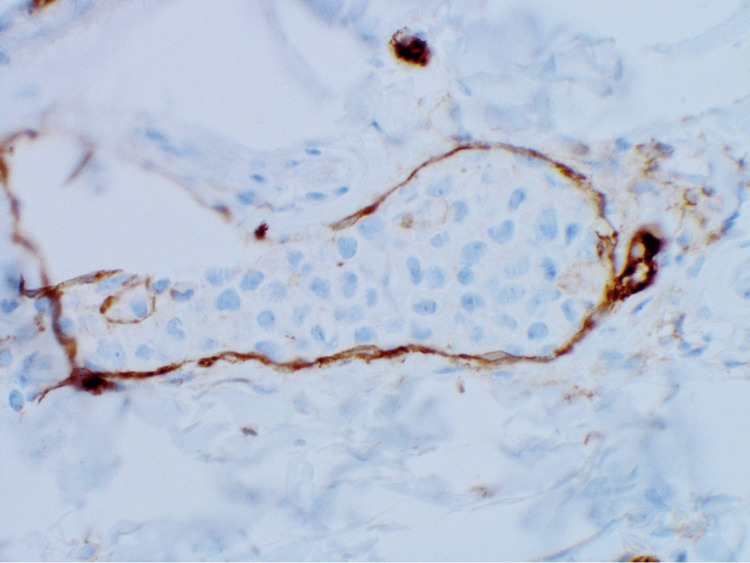
CD31 highlights the atypical lymphocytes and confirms the intravascular location (100×).

**Figure 7:**
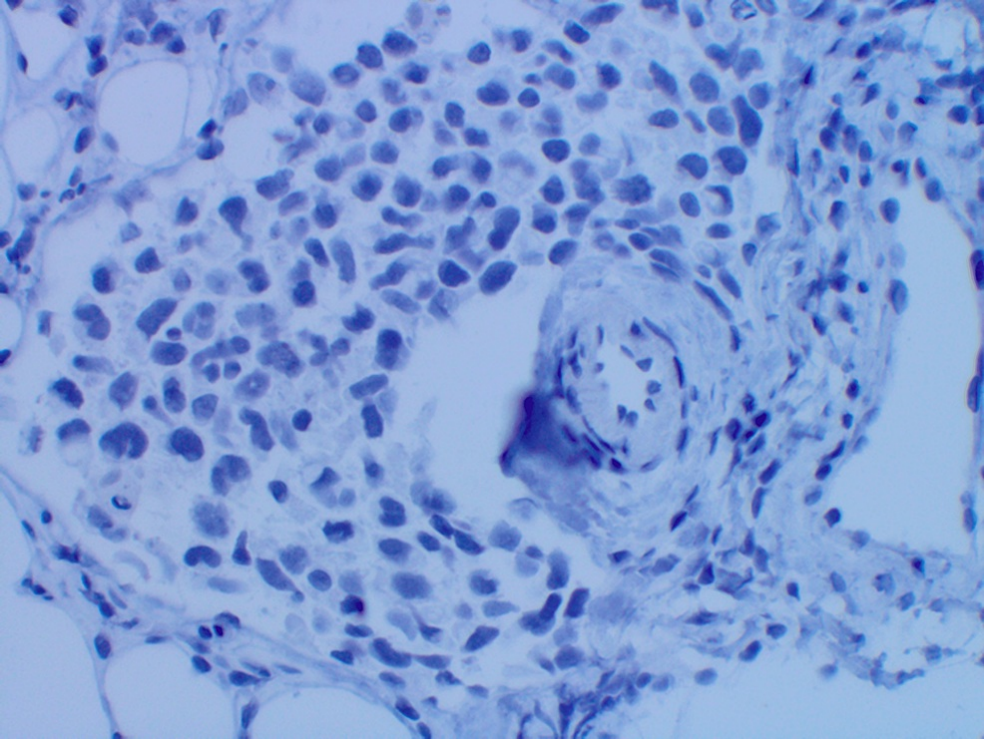
Immunohistochemistry confirming negative staining for anaplastic lymphoma kinase (100×).

Later, an erythematous patch adjacent to the prior subcutaneous nodule was noted at the time of suture removal. There was clinical suspicion of this new patch for another lymphoma. A 5-mm punch biopsy was performed, revealing an identical histopathologic diagnosis and immunohistochemistry findings as the first nodule. The second biopsy revealed multiple dilated ectatic vascular spaces containing large pleomorphic mononuclear cells within the dermis that were surrounded by a relatively bland-appearing lymphocytic inflammatory infiltrate. CD3 and CD30 were positive in the cells within the intravascular spaces. CD31 highlighted the cells were confined to the vascular spaces (Figure 6). ALK staining was negative, supporting a pathological diagnosis of primary C-ALCL (Figure 7). The patient was referred to hematology/oncology for further treatment and workup. A subsequent positron emission tomography scan was performed by oncology, which did not highlight any other areas of systemic involvement. Due to the patient’s age and localized nature, the oncologist and patient opted for continuous monitoring, with the possibility of radiation treatment in the future. After 13 months of monitoring, the patient returned to the clinic with two new firm nodules on the left chest. Based on his known history of lymphoma, there was clinical suspicion of recurrence. A singular nodule was excised, and histopathology revealed a nodular and diffuse infiltrate of lymphocytes filling the dermis but not confined to vascular spaces. Further workup was deferred to the patient’s treating institution.

## Discussion

Intravascular lymphoma is a rare form of lymphoma characterized by large, malignant cells within the lumen of the vasculature. Currently, the WHO classification system only identifies B-cell intravascular lymphoma as a distinct entity [2]. As such, most identified cases of intravascular lymphoma are of B-cell lineage and can be classified as systemic or primary cutaneous, with cutaneous-limited involvement showing a better prognosis [3]. Only rarely has a T-cell phenotype been documented (Table 1).

**Table 1:** Existing literature of intravascular anaplastic large cell lymphoma with primary cutaneous involvement. Information on the age/gender of the patient, treatment, and the immunohistochemical panel is provided.

Case	Age/Gender	Treatment	Immunohistochemical panel
Banafshay et al. (current report)	87/M	TBD	CD30+/CD3+/ALK-
Nguyen et al. (2015) [4]	61/F	Topical corticosteroids	CD30+/CD3+/ALK-
Metcalf et al. (2013) [5]	86/M	Local radiotherapy	CD30+/CD3+/ALK-
Iacobelli et al. (2012) [7]	39/F	Chemotherapy	CD30+/CD3-/ALK-
Wang et al. (2011) [6]	47/F	Surgery, chemotherapy	CD30+/CD3+/ALK-
Zizi-Sermpetzoglou et al. (2009) [8]	48/F	Surgery	CD30+/CD3+/ALK n/a

Nguyen et al. [4], Metcalf et al. [5], and Wang et al. [6] previously reported cases of intravascular primary C-ALCL. These cases shared a similar immunohistochemical panel with our case, with CD30 and CD3 positivity and ALK negativity. The patient described by Nguyen et al. [4] presented with a nodule on her anterior abdominal wall and was diagnosed with intravascular, cutaneous, CD8-positive ALCL. Her lesion resolved with topical corticosteroids, and the patient had been in clinical remission for 48 months at the time of reporting. Metcalf et al. [5] described a patient presenting with a left shin plaque that was also confirmed to be primary cutaneous intravascular ALCL. This patient was treated with local radiotherapy and had been in remission for seven months at the time of publication. The patient with intravascular ALCL described by Wang et al. [6] presented with several erythematous plaques and patches on her back. The lesions were surgically removed but later returned around her left breast. Three cycles of chemotherapy were performed, but the patient had a subsequent relapse. Iacobelli et al. [7] reported a case of a patient with a left posterior shoulder lesion. The female patient was treated with four rounds of cyclophosphamide, doxorubicin, vincristine, prednisolone, and 40 gray external-beam involved-field radiotherapy in 25 fractions. The patient remained in remission for seven months after the completion of treatment. Lastly, Zizi-Sermpetlogou et al. [8] described a patient with primary intravascular vulvar lymphoma of T-cell origin 13 years prior. However, additional follow-up information was not found.

## Conclusions

In summary, intravascular C-ALCL is an infrequent diagnosis. The prognosis is unclear whether this lymphoma behaves more aggressively, similar to other intravascular lymphomas, or behaves similarly to primary cutaneous CD-30-positive ALCL. Distinction from systemic ALK-negative ALCL is essential as the prognosis is unfavorable in systemic ALK-negative ALCL, and an appropriate staging workup is necessary to distinguish the two. Existing cases give us a glimpse into potential treatment options for the inflicted patients, but the efficacy of treatment and data on remission are limited. This uncertainty highlights the need to investigate additional cases to determine the appropriate treatment and clinical management of these patients.
